# Automatic Image Processing Algorithm for Light Environment Optimization Based on Multimodal Neural Network Model

**DOI:** 10.1155/2022/5156532

**Published:** 2022-06-03

**Authors:** Mujun Chen

**Affiliations:** College of Information Engineering, Henan Vocational College of Agricuture, Zhengzhou, Henan 451450, China

## Abstract

In this paper, we conduct an in-depth study and analysis of the automatic image processing algorithm based on a multimodal Recurrent Neural Network (m-RNN) for light environment optimization. By analyzing the structure of m-RNN and combining the current research frontiers of image processing and natural language processing, we find out the problem of the ineffectiveness of m-RNN for some image generation descriptions, starting from both the image feature extraction part and text sequence data processing. Unlike traditional image automatic processing algorithms, this algorithm does not need to add complex rules manually. Still, it evaluates and filters through the training image collection and finally generates image automatic processing models by m-RNN. An image semantic segmentation algorithm is proposed based on multimodal attention and adaptive feature fusion. The main idea of the algorithm is to combine adaptive and feature fusion and then introduce data enhancement for small-scale multimodal light environment datasets by extracting the importance between images through multimodal attention. The model proposed in this paper can span the semantic differences of different modalities and construct feature relationships between different modalities to achieve an inferable, interpretable, and scalable feature representation of multimodal data. The automatic processing of light environment images using multimodal neural networks based on traditional algorithms eliminates manual processing and greatly reduces the time and effort of image processing.

## 1. Introduction

The continuous progress and development of Internet technology have brought an unprecedented amount of data to human beings, and such a large amount of data must have hidden potential values. The popularity of various hardware terminals also allows mining the matter in a large amount of data, and these changes have promoted machine learning research. The principles related to machine learning are mainly penetrated in two fields: computer vision (CV) and natural language processing (NLP). Computer vision focuses on making computers “see” the world the way humans do, that is, making them understand images. Nowadays, computer vision has become inseparable from human life, such as cell phones scanning QR codes, face recognition, video surveillance, and processing remote sensing images. Natural language processing enables computers to communicate with humans through human languages, such as using machines for language translation and sentiment analysis of website comments. Developments in these two fields have been able to assist humans in various tasks and are still being refined to be used for more complex problems in the future. The increase in the amount of data makes the data more and more complex, and single information such as image, language, and sound is no longer separated. Still, more often, they are fused, so the study of multimodal data is gradually becoming a hot research topic for researchers. For artificial intelligence to better understand the world, it is necessary to improve the machine's ability to understand different forms of information.

Multimodal data covers various data types that people can access, including images, text, sound, and sensors. These data are not only naturally occurring but also abstract data types evolved by human beings in the long-term cognitive process, so, for multimodal data, each piece of modal data not only has a vast difference in the storage form but also has essential differences in the abstract semantic features. Therefore, for multimodal data, each piece of modal data has not only a huge difference in storage form but also a fundamental difference in abstract semantic features. Therefore, how to learn multimodal data features has been an essential topic of research in the field of data mining. Deep learning has proved its powerful feature extraction ability in several AI-related applications, so deep understanding has a vital role in feature learning in each modality, such as image feature extraction using a convolutional neural network to achieve image classification and speech feature extraction using a Recurrent Neural Network to acquire speech and text conversion. The ability of deep learning to learn features of a single modality is relatively mature. However, the modal components extracted by traditional deep learning feature extractors are uninterpretable, resulting in that the parts of multiple modalities cannot be well correlated. In addition, the features obtained by deep learning feature extraction are only the feature distribution of a large amount of data. Still, they cannot correspond with external knowledge and knowledge modeling, so deep learning cannot satisfy the relationship modeling and feature understanding between different modalities. For this reason, a more comprehensive multimodal feature learning model that can span semantic differences of other modalities and build feature relationships between different modalities is needed to seek a more comprehensive multimodal data referenceable, interpretable, and scalable feature representation. This paper proposes an automatic image processing algorithm for light environment optimization based on the multimodal neural network model. Based on the traditional algorithm, the multimodal neural network is used to automatically process the light environment image, eliminating the need for manual processing. It greatly shortens the time of image processing and saves time and effort.

The structure of this paper is as follows.

The first chapter is the introduction. This chapter summarizes the research background and significance of automatic image segmentation, research status, and existing problems and finally gives the research content, innovation, and structural arrangement of this paper.

The second chapter is related work. To provide a theoretical basis for subsequent algorithms based on fully convolutional neural networks, this chapter mainly introduces image segmentation related technologies, fully convolutional neural networks, and deep learning related theories.

The third chapter is the multimodal neural network model design. This chapter mainly introduces an improved automatic segmentation method of human images, which realizes the automatic segmentation of human images. In addition, this chapter locates the position of the character, assists the character segmentation network to segment the image of the character more accurately, and conducts experimental analysis and result evaluation of the algorithm.

The fourth chapter realizes the automatic image processing algorithm for optimization of light environment. This chapter mainly introduces the further segmentation of the character edge region based on the character segmentation network in the third chapter and proposes an improved edge segmentation algorithm, which is based on the closed demapping algorithm, and adds a global color space model and dynamic local optimization. Finally, the algorithm is experimentally analyzed.

The fifth chapter is the analysis of the results. The performance test of the multimodal neural network model and the simulation experiment of the light environment optimization image automatic processing algorithm are carried out.

The sixth chapter presents the conclusions. This chapter summarizes the research results of this paper, points out the deficiencies, and looks forward to future work.

## 2. Related Works

The MCP artificial neuron model emerged, which was the first model to apply the idea of neural networks. The model uses a computer to simulate the neuronal transmission and response of the human brain and divides the complex neuron into three parts: the input part is multiplied with values based on weights, the middle of the neuron is a linear combination of the output values of the upper part, and finally the activation process is at the end of the simulated neuron [1]. The perceptron algorithm was also proposed. The MCP neuron model was first used in the field of classification for machine learning. Multidimensional input data were binary classified by the perceptron algorithm [2]. The model was able to automatically optimize the network weights during the classification process because of gradient descent. The first wave of neural networks came, and, after several years of service and validation, scholars found that the model was able to converge [3]. Nowak's discovery brought neural networks to a standstill for almost two decades, in which Minsky demonstrated that the perceptron could only perform linear combinations of inputs [4]. The linear model could not accurately predict even the most specific heterogeneous problems when encountering nonlinear problems. The second wave of neural networks arrived, with the backpropagation algorithm for multilayer perceptrons being proposed, while the concept of deep learning was first introduced into machine learning. The algorithm used the Sigmoid function to increase nonlinear problems' capability. The third wave of neural networks stepped into the limelight in 2006, and, since then, more researchers have been conducting connected neural networks research. The research has become more in depth, and deep learning has developed rapidly.

*Threshold-Based Segmentation Method*. Among the many image segmentation methods, the most classic and vibrant application scenario is the threshold segmentation method, mainly because of its simple algorithm principle, easy implementation, stable performance, and high efficiency [5]. Threshold segmentation is essentially a comparison of the grayscale values of all pixels by selecting a feature threshold with differences and then using the comparison results to classify all pixels one by one to achieve the effect of segmentation. It can mainly handle images with significant grayscale differences between the target and the background. The most used method to achieve good segmentation results in single thresholding is the maximum interclass variance (Otsu) algorithm. In multithresholding, K-1 thresholds are used to separate K classes, that is, to calculate the variance between image classes. In terms of segmentation effect, multithresholding is more effective than single thresholding [6]. Therefore, determining the threshold value and the number of thresholds is directly related to the impact of good threshold segmentation. The function type of threshold selection can be divided into the maximum entropy method, interclass variance method, cross-entropy method, minimum error method, and fuzzy entropy method. Although the threshold segmentation method is widely used, it is vulnerable to noise interference. A network architecture based on a fully convolutional neural network (U-Net) was proposed and used for semantic segmentation of medical images by Ranneberger et al [7]. Noda K. et al. proposed null convolution [8]. The null convolution layer can enhance the corresponding receptive field index without reducing the spatial dimension. H. Li suggested using fully connected conditional random fields in spatial dimension to achieve a spatial pyramidal pooling scheme [9].

The continuous research on deep neural network algorithms has driven the implementation of deep learning in practical applications. Deep understanding has made remarkable development in the traditional computer vision image processing field and the emerging areas such as natural language processing and speech recognition [10]. The focus of this paper is the study of methods for multimodal feature fusion in remote sensing images on semantic segmentation. Visual learning, image processing, and information interpretation in the human brain are simple yet tricky tasks for machines to accomplish. The advent of the deep learning era has brought new solutions to computer vision image processing. Deep learning aims to obtain deep abstract features of an image, which is obtained by feature extraction of low-order information based on the image itself and then by operations such as convolution and activation functions [11]. These abstract features can accurately reflect the essential elements of an image. Take the image classification task as an example; traditional image classification methods generally use a support vector machine, K-nearest neighbor algorithm, and so forth. With the advent of deep learning, image classification accuracy based on convolutional neural networks has been significantly improved compared with traditional image classification methods [12].

## 3. Multimodal Neural Network Model Design

Convolutional neural networks (CNNs) are a class of feedforward neural networks that include convolutional computation and have a deep structure and are one of the representative deep learning algorithms. Convolutional neural networks are also known as “Shift-Invariant Artificial Neural Networks (SIANN)” because of their symbolic learning capability and their ability to classify input information shift-invariantly according to their hierarchical structure.

Graph convolutional neural networks are essential for graph structure data feature extraction. In multimodal feature learning graphs, neural networks are applicable not only to modeling topological relationship graphs within each modality but also to modeling topological relationships between multiple modalities. The graph neural network based on spectral analysis is one of the most common neural graph networks. Its central idea is the propagation of features of adjacent nodes, where the general expression for the propagation of feature messages can be expressed as follows:

(1) $$\begin{array}{c} h_{i}^{l+1}=\delta \left(\;\right.\overset{N}{\underset{j\in N_{i}}{AGGREGATE}}\left(h_{i}^{l},h_{j}^{l}\right). \end{array}$$

In (1), the feature vector of each node in the *l*th layer of the graph neural network is represented as *h*_*i*_^*l*+1^, *g*(*∗*) is the message mapping function, which is used to construct the message feature, and *N*(*i*) represents the set of adjacent nodes of the node. AGGREGATE means to aggregate the messages spread from different adjacent nodes, which can use cascade, maximum pooling, average pooling, and other functions, and *σ* means nonlinear activation function.

A graph convolutional neural network first requires the construction of a relational topology graph. Then the features of each node of the graph network are propagated through the adjacency relationship using the feature propagation algorithm. Finally, interfeature relationship extraction is performed. The feature representation output from the graph convolutional neural network is feature-extracted from the relational topological graph by pooling or cascading and so forth, to obtain abstract representations of the graph structure data at different levels [13]. The graph neural network can also be modified according to the task. In addition to graph convolutional neural networks, there are graph attention networks (GAT) and relational graph convolutional neural networks (R-GCN). These graph neural network structures aim to solve the feature extraction of graph networks with different designs and feature relations. In addition, graph neural networks can exploit features propagated by the characteristics possessed by the graph structure and migrate using the knowledge not seen by the introduced knowledge graph and thus have infertility and interpretability compared to traditional neural networks, which lays the foundation for building interpretable multimodal feature learning.

In the social image relabeling problem, the image dataset contains *m* images with *n* classes of social tags, and each print will correspond to several types of labels. The labeling relationship between *m* images *X*={*x*_*i*_}_*i*=1_^*m*^ and *n* classes of social tags *C*={*T*_*j*_}_*j*=1_^*m*^ forms a binary matrix *Y* ∈ {0,1}^*m*×*n*^. The element *y*_*ij*_ ∈ {0,1} in the *i*-th row and *j*-th column of the matrix indicates whether the *i*-th image *X*_*i*_ is labeled *T*_*j*_; when *y*_*ij*_=1, it indicates that the user has marked the *i*-th image *X*_*i*_*T*_*j*_, and a zero indicates that it is not labeled. The social image relabeling algorithm aims to find an ideal set of labeling matrices *Z* ∈ {0,1}^*m*×*n*^ that can correct the deficiencies of the original labeling matrix *Y* and accurately describe the image information. Matrix decomposition is a standard solution to this problem. It decomposes the social label matrix *Y* into two low-rank matrices *P*_*i*_ ∈ *R*^*m*×*t*^, *Q*^*n*×*t*^ such that *Y* ≈ *PQ*^*t*^. The element of the *i*-th row in *PP*_*i*_ ∈ *R*^*t*^ is the *r*-dimensional hidden feature of the image *X*_*i*_. Classical matrix factorization models train the model using the *L*2 loss function, which implicitly assumes that the noise matrix *E* follows a Gaussian distribution.

Similarly, the element *q*_*i*_ ∈ *R*^*t*^ in the *j*-th row of *Q* is the *r*-dimensional hidden feature of the label *T*_*j*_. There are various matrix decomposition methods for solving *P* and *Q* in different ways for different applications. The most basic form is the singular value decomposition (SVD).

(2) $$\begin{array}{c} {\left\|Y+PQ_{T}\right\|}^{2}+\left\|\frac{\gamma_{1}}{2}+F^{2}\right\|=\left\|\frac{\gamma_{1}}{2}Q_{F}\right\|,\\ AP\left(q\right)=\frac{L_{p}}{\sum_{i=1}^{n}p_{q}\left(r\right)}+1. \end{array}$$

The difference between the observation matrix *Y* and the ideal labeling matrix *Z* is the noise *E* present in the social labeling. The perfect labeling matrix *Z* can be deduced backward if the noise *E* can be accurately modeled. The classical matrix decomposition model uses the *L*2 loss function to train the model, implicitly assuming that the noise matrix *E* obeys the Gaussian distribution. However, Gaussian distribution is not necessarily the most appropriate noise assumption, and there may be better probability distributions to obtain better results for image relabeling [14]. By comparing five probability distribution assumptions, Gaussian distribution, Laplace distribution, Poisson distribution, *t*-distribution, and logistic distribution, and exploring the effect of social labeling noise, this section proposes the Corsi matrix decomposition method to generate better image annotation results by exploiting the robustness and excellent product of Corsi distribution in fitting social labeling noise. This section first derives the general probability matrix decomposition model, introduces five probability distribution assumptions, derives their formal representation, and introduces the distribution characteristics and finally introduces the Corsi matrix decomposition model in detail and verifies its effectiveness with experiments.

(3) $$\begin{array}{c} y_{i}=f\left(\sum_{i=1}^{n}w_{ij}x_{j}-b_{i}\right),\\ h_{t}=f\left(W_{xh}X_{t}-W_{xh}h_{t+1}\right). \end{array}$$

The semantic segmentation based on RGB-D multimodal images is mainly reflected in utilizing RGB images and Depth images. Nowadays, there are three common ways to combine them. The first is a single-branch structure, which directly stacks RGB and Depth images' channel information representation. It then uses them as inputs to the network to simultaneously extract feature information [15]. The network inputs are in the form of four-channel features with three channels of RGB, as well as a single channel of Depth in series or six-channel features with three tracks of RGB and three channels of HAA encoded depth information. The second one is by constructing a dual-stream network, in which RGB and Depth images are used as two branches of the network to first perform a feature extraction process without interfering with each other in the encoding stage, followed by fusing the feature maps of the two branches into one feature map in the decoding stage and finally performing a simultaneous training process of a single unit [16]. The third one is based on the second one with separate training and layer-by-layer fusion, in which two parallel networks are used to extract features separately and then the feature maps extracted from each layer are fused.

**Figure 1:**
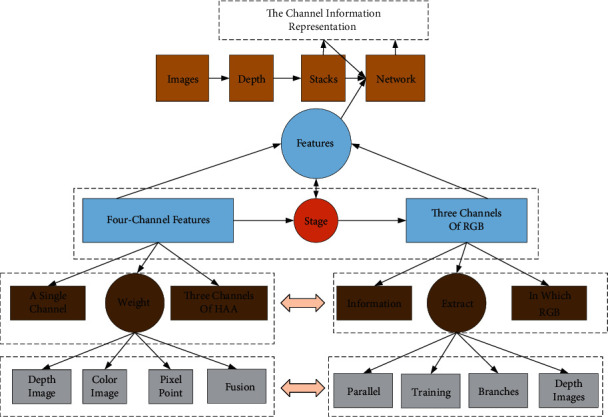
The multimodal neural network model.

Due to the difference in the information contained in Depth image and color image, Multimodal Complementary Attention (MCA) is proposed to perform feature extraction on any pixel point by first selecting the features of the two feature maps of RGB-D and assigning more significant weight to the essential elements that contribute more, and vice versa. A smaller weight is given. Since the feature points of the RGB image and Depth image are aligned one by one, the matrix of the two feature maps after weighting is summed. Finally, the fused image is added to the RGB-D fusion modality, which can supplement the general information of the RGB-D multimodal picture. As shown in Figure 1, a multimodal neural network model is shown. The left figure shows the fusion of RGB and Depth features of the first coding block, and the right figure shows the typical fusion of the three branches. The GAP module represents the global average pooling (GAP) layer, and the CBR module includes a convolutional layer Conv, a normalization layer BN, and a ReLU activation layer.

The shallow features of deep convolutional neural networks mainly extract more abstract detail representations, while the deep parts are represented as factual semantic information. In the external feature fusion in the coding stage, this paper uses the MCA module to perform weighted complimentary summation operations on the multimodal features. In contrast, for the modular feature fusion in the middle transition between coding and decoding, it is necessary to enhance the acquisition of high-level semantic information by RGB-D multimodal features and to solve the problem of aligning the two original RGB-D image elements by the nonlinear mapping of successive convolution in the early coding stage. Based on this, Multimodal Global Attention (MGA) is proposed in this paper.

(4) $$\begin{array}{c} B=\left[B_{1},B_{2},\text{…},B_{n}\right]\in R^{N\times H\times W},\\ j_{t}=\sum_{i=0}^{C}\exp\left(R_{i}^{t}\cdot G_{j}\right). \end{array}$$

## 4. Light Environment Optimization Image Automatic Processing Algorithm Implementation

With the rapid development of the industry, digital image technology has been widely used, and the field of video multimedia has developed rapidly. In the field of literature and art, the standard digital image technology is digital photography, post production, special effect production and editing, as well as game development, animation production, digital restoration of historical images, color restoration of black-and-white images, industrial design, etc., gradually forming a multimedia art culture dominated by computers. While digital image technology is developing rapidly and taking pictures is becoming more convenient, people are increasingly active in taking pictures of themselves or others. More photos of people are uploaded to the network [17]. As the number of images of people increases, people need to blur the background, replace the environment, change the tone of the foreground people, and so forth. We need to use a pen to draw a few lines to mark the human body in this software. Then the keying software automatically generates the foreground of the person, and when the segmentation effect is not satisfactory, you can continue to draw a few lines until the segmentation area is just wrapped around the human body; in some commercial software, it is often used like Grabcut keying algorithm for keying, the user draws a matrix box to frame the foreground of the person, and the software automatically calculates the segmentation [18]. This software requires a lot of human interaction, requiring the user to indicate where he wants to deduct the foreground, which is inefficient and cannot be processed quickly in batch when many images need to be keyed. Therefore, users need software that can automatically extract the human body from the picture, which will significantly facilitate image processing operations and improve the efficiency of image processing.

(5) $$\begin{array}{c} a_{i}\approx \sum_{j=i}a^{j}I_{i}^{j}-b. \end{array}$$

**Figure 2:**
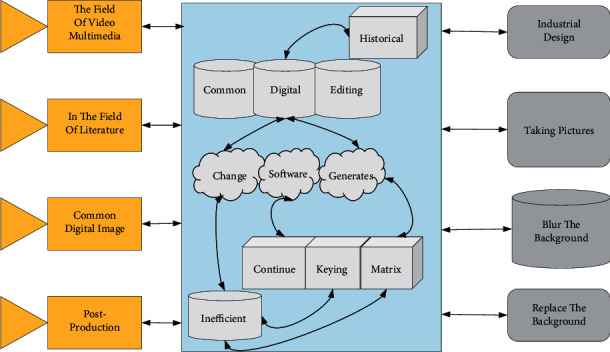
Flow of automatic image processing system.

The overall process of the automated image processing system is shown in Figure 2. After opening the software, the user is first prompted to input a picture of a person, enter it into the system to get the foreground of the person, and filter. Then the system automatically optimizes the edges to get a precisely segmented foreground of the person and then displays only the foreground of the person and prompts the user to modify or export optionally. The system provides the option of cartoonizing the image of the person. The user can choose to cartoonize the foreground of the person and export it to generate a picture containing only the foreground of the person.

*Qt* Development Framework is a cross-platform development framework developed by a Norwegian software company, the famous KDE (Linux system popular software) developed for *Qt*. *Qt* provides a layer of abstraction to achieve the concept of a code running on various machines, supporting Windows, Linux, Mac, and embedded Linux. *Qt* for *Python* 12-741 is *Qt*'s interface to the *Qt* runtime environment for *Python*, which allows you to use the *Python* language to call *Qt*'s core libraries, UI graphical user interface, and so forth. Although *Python* initially supports cross-platform, its GUI support is poor. With *Qt*'s GUI interface, *Python*'s cross-platform interactivity is greatly enhanced.

The core modules of *Qt* are the graphical user interface, the web application interface, Webkit (which provides the HTML rendering engine), *Qt* Creator, and the signal and slot mechanism. *Qt*'s signal and slot mechanism, instead of callback operations, when the user clicks a button, hovers over a component, drags and drops, and so forth, will be sent to the receiving slot in the form of a signal.

Graph-cut algorithm is a commonly used image energy optimization algorithm and is a widely used image segmentation algorithm. Image segmentation can be considered a kind of image classification problem, where foreground pixels are denoted as class *O*, and background pixels are represented as class *B*. The best case for image segmentation is that the graph-cut boundary is at the edge of both the foreground and background. Let *L*={*l*_1_, *l*_2_, *l*_3_,…, *l*_*p*_}, where *p* is the number of pixels and *l*_*p*_ ∈ {*O*, *B*}; that is, any pixel can be classified as foreground or background.

(6) $$\begin{array}{c} E\left(L\right)\alpha^{2}R\left(L\right)-\alpha B\left(L\right). \end{array}$$

**Figure 3:**
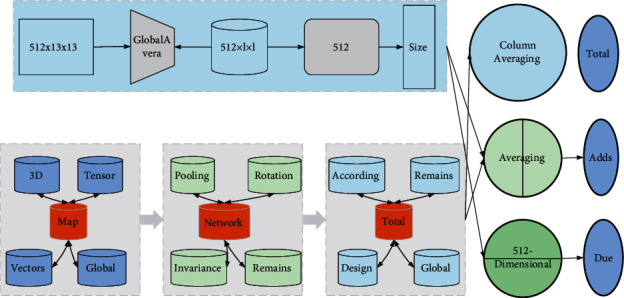
Image feature extraction.

The number of samples collected in this project is only 64 cases. Due to the small number of pieces, if the commonly used convolutional neural networks (such as ResNet) are selected, the quality of the extracted image features may be affected by overfitting or model nonconvergence due to the large size of these models and too many parameters [19]. Therefore, a convolutional neural network with a smaller model must be selected. Thus, the final solution uses the SqueezeNet convolutional neural network with fewer parameters to extract image features. The size of the extracted image components is related to the network structure, and, according to the location of the features extracted in SqueezeNet, two different parts can be advanced to 512-dimensional column vectors and 2 × 13 × 13 tensor, respectively. The size of the output of different layers of SqueezeNet varies. In this scheme, two types of essential features of suitable size are extracted, namely, 512 × 13 × 13 features and 2 × 13 × 13 features. Since 512 × 13 × 13 parts have too many parameters, global average pooling (GAP) is used to compress the features into 512 × l × l, that is, 512-dimensional column vectors, as shown in Figure 3. The global averaging pooling adds up the parameters of each channel and then averages them; the number of pipelines remains the same before and after the transformation, while the dimension of each channel is changed from the original two-dimensional matrix to a numerical value. Therefore, the global average pooling operation can transform the 3D tensor feature map output from the convolutional neural network into features in column vectors (component vectors). Global averaging pooling compresses the number of parameters while maintaining invariance of properties such as rotation translation and is a standard method used in the design of convolutional neural network structures.

**Figure 4:**
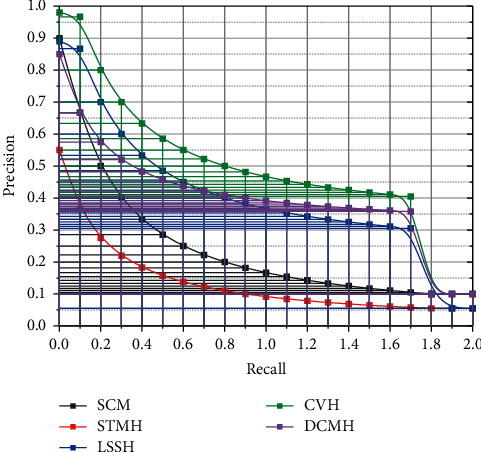
Precision-recall curve of image automatic processing algorithm.

The NYU Depth V2 dataset contains 25,000 instances collected from the social photography site Flickr. Each image is tagged with 38 semantic concepts and some associated text labels and manually annotated using at least one of the 24 category tags. Our experiments selected 20,015 data points with no less than 20 text tags. The dimensionality of the feature vector for the images in this dataset is 150 dimensions, and a 50-dimensional feature vector represents the text. The NUS-WIDE dataset is a huge dataset composed of web images collected from reality, including 269,648 instances and images with associated text tags. There are 81 basic factual concepts available for manual annotation for retrieval evaluation [20]. The dataset was first preprocessed before using this dataset as the algorithm's input. Ten standard labels were selected as the annotation of the images, and the rest of the data were removed from the dataset, so we obtained 186,577 image-text pairs for the experiment. To evaluate the effectiveness of our proposed method, we compared it with five state-of-the-art multimodal hashing methods: SCM, STMH, LSSH, CVH, and DCMH. The exact recall curves of all methods on both datasets are shown in Figure 4. We found that our method is more effective than the other methods through the comparative analysis.

For the MIRFLICKR-25K dataset, we randomly selected 10,000 instances as the training set. We used 2,000 instances of this dataset as the test set and the rest as the retrieval set for testing. We randomly sampled 10,500 data points for the NUS-WIDE dataset as the training set. We used 2,100 instances of this dataset as the test set and the rest as the retrieval set for testing. Alexie network has been pretrained on the ImageNet dataset and fine-tuned while training our model. In our experiments, we set *y* = 0.3 and *u* = 0.1. In addition, the batch size is fixed at 128, and the algorithm can be run 500 times.

## 5. Analysis of Results

### 5.1. Multimodal Neural Network Model Performance Tests

**Figure 5:**
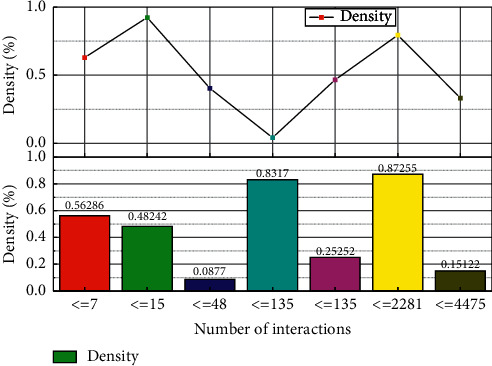
Model sparsity test results on NYU Depth V2 dataset.

**Figure 6:**
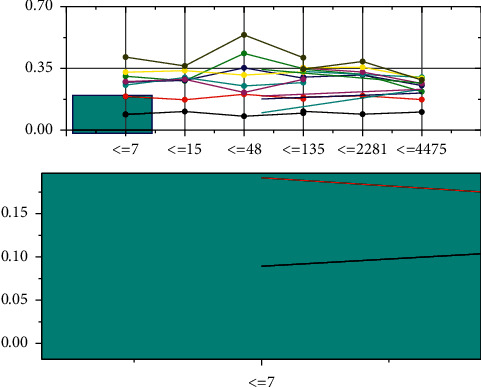
Model sparsity test results on SUN RGB-D dataset.

As shown in Figure 5, according to the test results of each model on the datasets NYU Depth V2 and SUN RGB-D, the models based on multimodal neural networks are better than the traditional recommendation algorithms. The advantage is more obvious when the user interaction is more sparse, but the advantage of KGAT is weakened when there are more user interaction records [21]. At the same time, the KGMRCF proposed in this chapter can maintain its advantage when there are more user interaction records. KGMRCF can maintain its advantage when there are more user interaction records. When testing the dataset Last-FM, the experimental results in Figure 6 show that the knowledge graph recommendation model and most models have similar trends in data with different sparsity. Since Last-FM is a music recommendation dataset, the recommendation results show that the accuracy of the model recommendation is higher for users with fewer interactions, which is because people generally use music attributes as the primary recommendation basis when they consume less music [22]. However, because this dataset has more interaction data and fewer knowledge graph relationships than the other two datasets, the KGMRCF model with multirelationship convolutional neural network for data with high sparsity is significantly better than the KGAT model, which also indicates that the KGMRCF model has better extraction ability for implicit collaborative relationship features with better extraction ability.

For both NYU Depth V2 and SUN RGB-D datasets, the performance indexes obtained by the model with a one-layer relational graph convolution network are better, which indicates that these data have fewer high-order implicit relational features. In contrast, the use of multilayer relational graph convolution will lead to the overfitting of the model. Then the model performance decreases, while, for the Yelp2018 dataset, there are more layers of relational graph convolution. On the one hand, this is because the knowledge graph of this dataset has a low overlap rate of relational feature subgraphs, which cannot map each entity to a common relational space through first-order multirelational feature propagation. On the other hand, there are more users' implicit relational preferences in the dining dataset, so the more layers of relational graph convolution, the better the extraction of higher-order multirelational features and the better the model performance.

**Figure 7:**
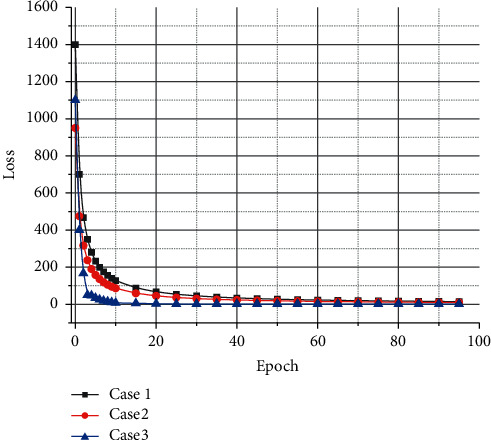
Comparison of results of training with different programs.

If the model is split and deployed separately on two servers in a distributed environment and no alternate execution policy is used, the iteration cycle takes about 1.08 seconds. The waiting time includes the execution time of the fully connected module and the time to synchronize the data to the fully connected module. The convolutional module slows down the process by creating GPU idle time while waiting for the remote fully connected module to return data [23]. If the alternate execution strategy is used, the time for intermediate operations is significantly longer, but, during this time, another iteration of the data is completed. Although the total time to run an iteration is about 1.69 seconds, in this iteration, essentially two batches of data are trained, resulting in an average cycle time of approximately 0.845 seconds per iteration, which is slightly faster than Caffe. Thus, it can be argued that the alternate execution strategy allows the time spent waiting for the fully connected module data to be covered entirely in the convolutional module of the distributed environment, thus guaranteeing the utilization of the GPU. Six fully connected processes and parameter centers are running in GPU 0, but there is still a surplus of computational power, so GPU 0 is used between 80% and 90%. In GPU {1–3}, each GPU runs two convolutional processes, and, according to the above analysis, the GPU can keep running at total capacity. Thus, its utilization remains almost 100%, which confirms the previous derivation. Therefore, Wheel can increase the computational power by one share for each additional GPU, as shown in Figure 7, achieving an approximately linear performance increase.

### 5.2. Light Environment Optimization Image Automatic Processing Algorithm Simulation Experiments

In addition to the three evaluation metrics of automatic image annotation models, namely, average accuracy, average recall, and overall performance, *F*1, Peak Signal-to-Noise Ratio (PSNR), Structural Similarity (SSIM), and Frechet Inception Distance (FID) Score are evaluation metrics to investigate the quality of image samples generated by CycleGAN-based data expansion method. The data samples expanded by CycleGAN outperform the data samples developed by random interception and noise perturbation methods in three evaluation metrics: PSNR, SSIM, and FID. Unlike PSNR and SSIM, FID uses the Inception network to extract image features. It then calculates the distance between the original image and the expanded image, so FID is evaluated from the perspective of convolutional features. The CycleGAN-based data expansion method performs complex operations but retains many essential features. The traditional data expansion method operates on the original image. Based on its simple algorithm principle, easy implementation, stable performance, and high efficiency, people choose the threshold segmentation method. The essence of threshold segmentation is to select different feature thresholds, compare them with the gray values of all pixels, and then use the results of the comparison to classify all pixels one by one, to achieve the effect of segmentation.

In contrast, the CycleGAN-based data expansion method first extracts the image features. It then reconstructs the image based on the extracted image features, in which the detailed parts of the original image are ignored. In contrast, the key elements are retained, making the reconstructed generated image more conducive to training a high-performance convolutional neural network. This makes the reconstructed image more conducive to training a high-performance convolutional neural network. The experimental results of deformable convolution and Batch Normalization on network performance are shown in Table 1.

The experimental comparison in Table 1 shows that the improved VGG19 network performs better. The improved VGG19 network in Scheme 3 improves 7.2% in average accuracy, 8.5% in the average recall, and 8% in comprehensive performance F compared with the original VGG19 network, indicating that the improved scheme in this chapter can improve the labeling performance of the network. The network performances of Scheme 3 and Scheme 4 are the same, and replacing the convolution with the deformable convolution will increase the number of parameters of the network. Therefore, Scheme 3is chosen as the best network structure. From Figure 7, it can be analyzed that, with the increase of training times, the loss value of Scheme *l* decreases steeply, while the loss value of Scheme 3 decreases gently. The loss value of Scheme 3 is stable when the training reaches 110 batches, while the loss value of Scheme 1 still has a decreasing trend and reaches saturation when the activity goes 130 packs. The analysis of the above experimental results shows that reducing the number of parameters in the fully connected layer and adding Batch Normalization can make the training process more stable and significantly reduce the training time of the network.

The segmentation results of RX-Net and RAX-Net proposed in this paper are closest to the actual labels. This demonstrates the effectiveness of the network structure, and the problem of semantic conflict can be effectively avoided by the multifeature extraction structure for multimodal images separately. The segmentation result of RX-Net is slightly inferior to that of RAX-Net, which is mainly because the noise in the picture makes the network misjudge the class of pixels. With the addition of the multiscale attention module, the segmentation results are significantly improved. This indicates that the multiscale attention module combined with multiscale features can provide a more comprehensive understanding of brain tumors' global and local information and enhance the weight of pixels in task-related regions based on this information. This also enables the network to determine the category attribution of pixels and suppress the interference caused by noise to the segmentation task more accurately.

**Figure 8:**
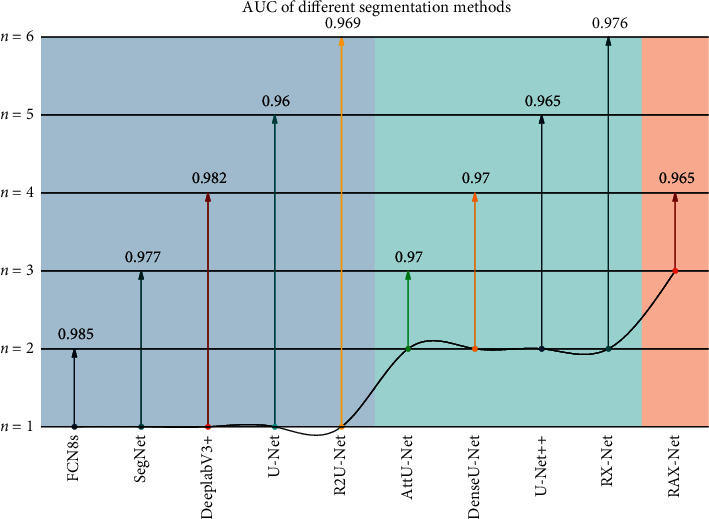
ROC curves and AUC for different segmentation methods.

Therefore, how to learn multimodal data features has always been an important research topic in the field of data mining. In many artificial intelligence-related applications, deep learning has proven its powerful feature extraction ability, so deep learning plays a very important role in feature learning in various modalities. The Receiver Operating Characteristic Curve (ROC) is a tool used to measure the nonequilibrium nature of classification. The ROC Curve and the Area Under the Curve (AUC) are often used to evaluate the merits of binary classifiers. The ROC curves and AUC of different segmentation methods are shown in Figure 8. The area on the right side of the figure results from the enlarged red box on the left side. It is clear from Figure 8 that RAX-Net obtains the best curve and AUC values for both, which further demonstrates that RAX-Net has the best segmentation performance.

Recommendation model training time is an essential indicator of model iteration and efficiency of new data used in recommendation systems. Since the iterative update of users' preferences and products is time-sensitive, it is essential to speed up model training to improve the recommendation accuracy of the model in practical applications, and having a high model training efficiency can improve the mining ability of users' short-term preferences and thus enhance user experience. Since the model training time is not only related to the computational complexity of the model but also related to the convergence time of the model, in general, the relational graph convolutional network takes more time than the graph convolutional network, so the model with more layers of relational graph convolution is chosen to have a longer model training time compared to the model with fewer layers.

**Figure 9:**
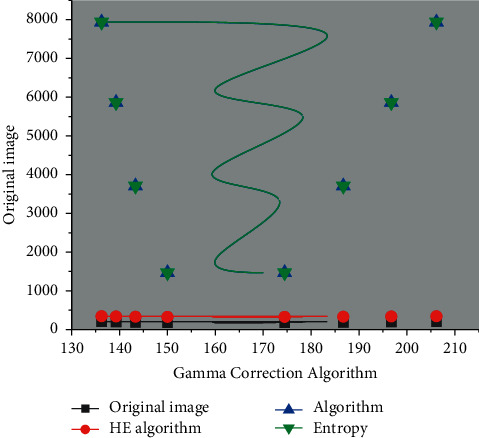
Image data comparison of processing results of different algorithms.

The image quality after processing by the algorithm in this paper has improved better, as shown in Figure 9. It is mainly shown that the value of standard deviation becomes larger, reflecting the enhanced contrast of the image; the importance of the average gradient becomes larger, indicating that the picture becomes more apparent; the value of entropy becomes larger, representing that more information can be extracted from the corrected image, which shows that the algorithm of this paper has achieved better results in preprocessing the image under complex lighting environment. With the continuous penetration of technology in daily life, the application of computer vision has become inseparable from human life, such as scanning QR code with mobile phones, face recognition, video surveillance, and processing remote sensing images.

In this paper, after processing the images with uneven illumination, the subjective visual effect shows that the equalization algorithm results in severe color distortion and overenhancement. The Gamma correction algorithm is better than the histogram equalization algorithm. Still, the shadow of the face in the image is not effectively handled, and the Gamma function is not adaptive enough in the value method. The algorithm in this paper deals with the problem of uneven illumination in the image effectively and has good color retention of the image and can correct the unevenly illuminated vision adaptively. To further compare the processing effects of different algorithms, this paper uses objective indicators such as standard deviation, average gradient, and information entropy to measure different algorithms. The standard deviation reflects the contrast characteristics of the image, and the average rise is an essential issue for image sharpness. An image preprocessing optimization algorithm is designed and implemented based on adaptive Gamma correction for complex lighting environments.

## 6. Conclusion

This paper designs and implements a multimodal neural network-based automatic image processing algorithm for image optimization in light environments with uneven illumination. Simulation experiments show that the algorithm in this paper effectively enhances the contrast, sharpness, and information of images in a fair environment and has good use in attenuating the effect of a complex light environment on the image being accurate. By conducting experiments on NYU Depth V2 and SUN RGB-D datasets, the model proposed in this paper has high automatic processing accuracy and shows some advantages in objective evaluation. Most multimodal neural network-based image segmentation methods require a large amount of labeled data for supervised learning. However, supervised learning methods will be brutal to accomplish the corresponding tasks for images where it is difficult to collect a large amount of labeled data. Due to the limited ability and time of the author, the automatic segmentation of human images and the automatic processing system of human images proposed in this paper still have many areas worthy of optimization, and there is still a lot of research space and development work in the future. For the automatic segmentation of human images, it is necessary to optimize the existing automatic segmentation system of human images, mainly from two perspectives: one is to continue to improve the segmentation accuracy, and the other is to speed up the segmentation speed and reduce the amount of segmentation calculations.

## Figures and Tables

**Table 1:** Evaluation results of each program.

Experimental program	P (%)	R (%)	*F*1 (%)
Plan 1	33.6	28.4	33
Plan 2	31	34.8	33.8
Plan 3	28.8	34.2	34.1
Plan 4	32.5	37.7	28.7

## Data Availability

The data used to support the findings of this study are available from the corresponding author upon request.
